# A Novel Dual-Emission Fluorescence Probe Based on CDs and Eu^3+^ Functionalized UiO-66-(COOH)_2_ Hybrid for Visual Monitoring of Cu^2+^

**DOI:** 10.3390/ma15227933

**Published:** 2022-11-10

**Authors:** Jie Che, Xin Jiang, Yangchun Fan, Mingfeng Li, Xuejuan Zhang, Daojiang Gao, Zhanglei Ning, Hongda Li

**Affiliations:** 1 College of Chemistry and Materials Science, Sichuan Normal University, Chengdu 610068, China; 2 The Experiment Center, Shandong Police College, Jinan 250014, China; 3 Liuzhou Key Laboratory for New Energy Vehicle Power Lithium Battery, School of Electronic Engineering, Guangxi University of Science and Technology, Liuzhou 545006, China

**Keywords:** metal–organic frameworks, probe, fluorescent, CDs, copper ions

## Abstract

In this work, CDs@Eu-UiO-66(COOH)_2_ (denoted as CDs-F2), a fluorescent material made up of carbon dots (CDs) and a Eu^3+^ functionalized metal–organic framework, has been designed and prepared via a post-synthetic modification method. The synthesized CDs-F2 presents dual emissions at 410 nm and 615 nm, which can effectively avoid environmental interference. CDs-F2 exhibits outstanding selectivity, great sensitivity, and good anti-interference for ratiometric sensing Cu^2+^ in water. The linear range is 0–200 µM and the limit of detection is 0.409 µM. Interestingly, the CDs-F2’s silicon plate achieves rapid and selective detection of Cu^2+^. The change in fluorescence color can be observed by the naked eye. These results reveal that the CDs-F2 hybrid can be employed as a simple, rapid, and sensitive fluorescent probe to detect Cu^2+^. Moreover, the possible sensing mechanism of this dual-emission fluorescent probe is discussed in detail.

## 1. Introduction

As one of the essential trace transition metals in organisms, copper ions (Cu^2+^) are widely distributed in the environment for various life activities [1]. For the human body, Cu^2+^ is required for cellular respiration, bone formation, and cardiovascular disease prevention [2]. However, excessive amounts of Cu^2+^ cause drowsiness, elevated blood pressure, liver damage, acute hemolytic anemia, neurotoxicity, and neurodegenerative diseases [3,4]. In recent years, multiple analytical techniques have been applied to the quantitative analysis of Cu^2+^, such as atomic absorption spectrometry [5], colorimetric methods [6], electrochemical techniques [7], and inductively coupled plasma emission spectrometry [8]. However, expensive instruments, tedious operation, long reaction times, and the need for trained operators greatly limit their application [2]. Hence, developing a facile, fast, and reliable method for the detection of Cu^2+^ in aqueous solutions is exceptionally significant.

Compared with traditional analytical methods, fluorescence technology has attracted the attention of researchers because fluorescence measurements are generally low-cost and have fast detection speed, high sensitivity, and easy visualization [9,10,11]. At present, fluorescent probes have been widely investigated for detecting Cu^2+^ [12,13]. Among them, metal–organic frameworks (MOFs) have aroused considerable interest owing to their flexible and adjustable structure, porosity, and large specific surface area, which is beneficial for concentrating trace amounts and enhancing the contact area between the probe and the analyte [14,15,16,17]. Wang et al. designed the fluorescent probe AuNCs/ZIF-8, which exhibited fluorescence turn-off responses to Cu^2+^ in the concentration range of 2–15 μM with a detection limit of 0.9 μM [18]. Jiang et al. successfully synthesized a PPN probe based on a natural β-pinene derivative nopinone, and the color of the solution could be observed to change from colorless to yellow after adding Cu^2+^ [19]. However, these fluorescent probes are based on a single emission, which makes the detection accuracy susceptible to environmental conditions including temperature, viscosity, pH, and operations. In contrast, the dual-emission ratiometric fluorescence probes can achieve good self-calibration in detection processes by measuring the fluorescence intensity ratio at two different wavelengths as a signal parameter [20,21,22]. Due to the fact that the measured fluorescence ratio signal is not influenced by the instrument and environment, the ratiometric fluorescent probes have high sensitivity, selectivity, and linear range. In addition, the dual-emission ratiometric probes have distinguishable visible color changes, which is greatly helpful for fast and on-site measuring by the naked eye. Thus, rationally designing and developing a MOFs-based ratiometric fluorescent probe with the improvement in detection performances is highly desirable.

In this paper, we report a novel fluorescent probe based on CDs and Eu^3+^ functionalized UiO-66 through a post-synthetic modification method (Scheme S1). The UiOs series material is an octahedral cage conformation MOFs material formed by ligating organic ligands with metallic zirconium as the metal center. UiO-66 is one of the more common materials in the UiOs series. The CDs-F2 hybrid exhibits a typical blue-emitting behavior from CDs and a clear red-emitting of Eu^3+^. CDs-F2 composite shows outstanding fluorescence properties for sensitive detection of Cu^2+^ in aqueous solutions. The results indicated that CDs-F2 can rapidly and sensitively detect Cu^2+^ in “on-off” mode. Moreover, the CDs-F2 film was made with a silicon plate, realizing precise visible detection by the distinguishable fluorescence color change. The work in this paper may provide an effective and intuitive method for the rapid detection of Cu^2+^ in water.

## 2. Experimental Details

### 2.1. Reagents and Instruments

All measurements were performed at room temperature. All chemical reagents and solvents are commercially available and were used directly without further purification.

The instruments and characterization are identical to our reported works [16,23].

### 2.2. Synthesis of UiO-66-(COOH)_2_ (Denoted as F1)

F1 was prepared according to the previous literature with some modifications [24]. The reactants, including ZrCl_4_ (1.0600 g), 1,2,4,5-benzenetetracarboxylic (H_4_btec, 0.1260 g), and p-Phthalic acid (PTA, 0.4989 g), were dispersed in a mixture of DMF (50 mL) and acetic acid (5 mL) at room temperature and further sonicated for 30 min. Then, the well-mixed liquid was heated in an oven at 160 °C for 24 h. After cooling, the as-obtained white solid was separated by centrifugation and washed with distilled water and methanol. In order to remove residual DMF from the samples, the solids were suspended in 30 mL of acetone for one week and the acetone solution was changed daily. Finally, the product was recovered in a vacuum at 70 °C.

### 2.3. One-Pot Synthesis of CDs@Eu-UiO-66(COOH)_2_ (CDs-F2)

CDs were synthesized on the basis of previous studies [25]. CDs-F2 was prepared by a one-pot post-synthesis modification. The mixture of 200 mg F1 and 0.04 M Eu(NO_3_)_3_·6H_2_O in 25 mL of CDs was stirred at room temperature for 24 h. Subsequently, the hybrid product was obtained by centrifugal washing and dried under vacuum at 70 °C.

### 2.4. Luminescence Sensing Experiments

Fluorescence detection of metal ions in water was carried out at room temperature. A total of 2 mg CDs-F2 is dispersed into distilled water (3 mL) and conduct ultrasound for 30 min, then 1 mL of different metal ions (0.01 M) was added into the dispersion (M^n+^ = K^+^, Mg^2+^, Cd^2+^, Co^2+^, Cr^3+^, Sr^2+^, Mn^2+^, Ni^2+^, Ca^2+^, Cu^2+^). Finally, their fluorescence spectra were collected.

### 2.5. Preparation of Fluorescent Films

The slide with dimensions of 10 mm × 25 mm × 1 mm was washed alternately with ethanol and distilled water and then put at room temperature to dry. The silica gel solution was obtained by adding 100 mg of sodium carboxymethylcellulose to 20 mL of distilled water, stirring until dissolved, and then adding 7.5 g of silica gel with continuous stirring. The silica gel solution was applied evenly on the dry slides, dried naturally, and then heated in an oven at 60 °C for 30 min to obtain silica gel plates. A total of 150 µL of CDs-F2 suspension was disposed of on the silica gel sheets and dried at 60 °C for 3 h to obtain the silica film sample.

## 3. Result and Discussion

### 3.1. Optimization of the Fluorescence Properties 

The photoluminescent (PL) properties of F1, Eu-UiO-66-(COOH)_2_ (denoted as F2)_,_ CDs, and CDs-F2 have been investigated in detail at room temperature. F1 shows a broad emission band peaked at 410 nm in the blue region (Figure S1a), resulting from π-π* transitions of the ligands [26]. Subsequently, a series of F2 samples were prepared by introducing different starting doping amounts of Eu^3+^ into the F2 compound. The emission spectra were collected as presented in Figure S1b. Except for the wide emission band centered at 410 nm, F2 exhibited several new emission peaks at 592 nm, 615 nm, 652 nm, and 700 nm, which were attributed to the ^5^D_0_ → ^7^F_J_ (J = 1–4) transitions of Eu^3+^ [27]. Both the emission of the ligand and emissions of Eu^3+^ appear simultaneously, indicating that Eu^3+^ has been successfully incorporated. In response to the increase in Eu^3+^ concentration, the emission intensity of Eu^3+^ remains much weaker than the emission intensity of the ligand, which indicates that the energy transfer effect between the organic ligand and Eu^3+^ is ineffective.

Considering the excellent physicochemical stability and abundant surface functional groups of CDs, we introduced CDs as a guest molecule to form an effective energy transfer to enhance the luminescence efficiency of Eu^3+^. The excitation and emission spectra of the blue-emitting CDs are shown in Figure S2a,b. The excitation spectrum displays two strong bands centered at 250 nm and 360 nm, respectively. Depending on the different excitation wavelengths, the emission spectra of CDs revealed different intensities, while the shape and profile for the emission peak changed little. The maximum emission peak at 430 nm of the prepared CDs was mainly caused by the surface state defects rather than by the eigenstate emission and their synergistic effect [28]. Moreover, different concentrations of Eu^3+^ were added to the CD solution. Figure S2c,d reveal that the emission intensity of CDs gradually decreased with the increase in Eu^3+^ concentration under the excitation at 360 nm or 250 nm UV light, which supported the presence of energy transfer between Eu^3+^ and CDs. However, the characteristic emission of Eu^3+^ was not observed. Such a phenomenon is caused by the high-energy vibrational coupling of Eu^3+^ with -OH in water, leading to the quenching of fluorescence belonging to Eu^3+^ [29]. Therefore, a rigid environment is needed to reduce the energy loss of the Eu^3+^ nonradiative transition.

**Figure 1:**
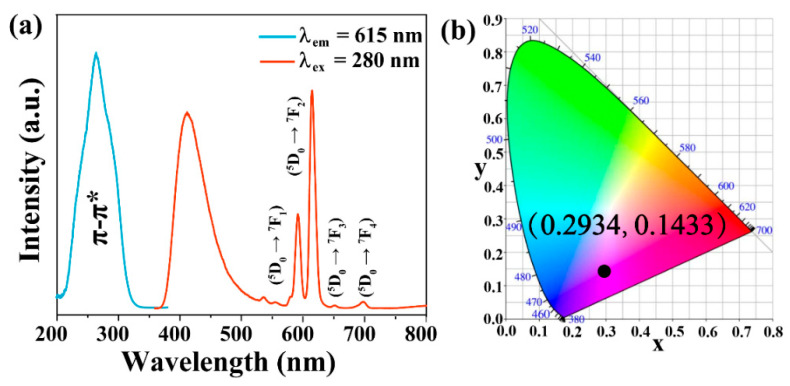
(**a**) The fluorescence of CDs-F2 and the screening of the excitation wavelength of the sample. (**b**) The corresponding CIE chromaticity diagram of CDs-F2.

Afterward, Eu^3+^ and CDs were introduced simultaneously into the matrix material F1. Excitingly, from the emission spectra of the synthesized CDs-F2 samples (Figure S3), both the red characteristic emission peak (615 nm) attributed to the Eu^3+^ and CDs’ blue characteristic emission peak (410 nm) can be observed. The strongest emission peak of the CDs is blue-shifted (from 410 nm to 430 nm). This phenomenon may be caused by the transformation of CDs from solutions to composite powders [30]. The characteristic emission intensity of CDs and Eu^3+^ changes with the starting doping amounts of CDs and Eu^3+^. Eventually, when the CDs and Eu^3+^ are tuned to 25 mL and 0.04 M, the characteristic emission intensity of CDs and Eu^3+^ basically reached a relatively balanced state (I_410 nm_/I_615 nm_ ≈ 1). This condition was selected for subsequent studies. To obtain the excitation wavelength of the material, we recorded the excitation and emission spectra of the material. The maximum excitation bands of the material appeared at 287 nm and 264 nm with monitoring wavelengths of 410 nm and 615 nm, respectively (Figure S4a). The emission spectra of the materials were measured at different excitation wavelengths in the range of 260–310 nm. It can be observed that with the increasing excitation wavelength, the emission intensity of Eu^3+^ gradually decreases, while that of CDs firstly increases and then decrease (Figure S4b). Given that the emission intensities at 410 nm and 615 nm are similar to the intensity when excited at 280 nm, 280 nm was adopted as the optimal excitation wavelength for the CDs-F2 hybrid. On the basis of those factors mentioned above, under the optimization of addition content (CDs: 25 mL and Eu^3+^: 0.04 M) and excitation wavelength (280 nm), the fluorescent excitation and emission spectra of the CDs-F2 are presented in Figure 1a, and the hybrid exhibits a reddish-purple color under the UV lamp with a CIE chromaticity coordinate of (0.2934, 01433) (Figure 1b). Therefore, this CDs-F2 with blue and red emission was explored as a dual-emission ratiometric fluorescence probe for the detection of metal ions.

### 3.2. Characterizations 

**Figure 2:**
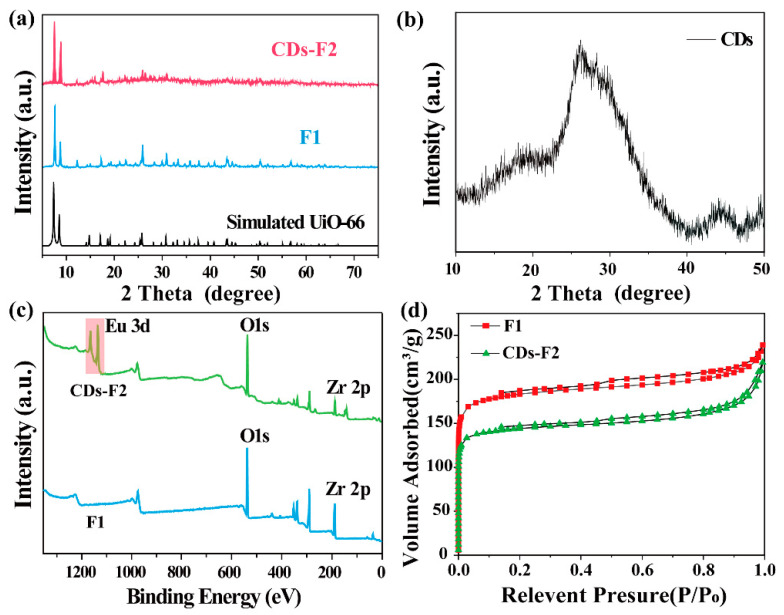
(**a**) PXRD patterns of simulated UiO-66 and as-prepared F1, CDs-F2, and (**b**) CDs. (**c**) XPS spectra of F1 and CDs-F2. (**d**) The N_2_ adsorption isotherms of F1 and CDs-F2.

The composition and crystal structure of the as-obtained products were studied by powder X-ray diffraction (PXRD) (Figure 2a). It can be seen that the XRD pattern of the prepared F1 and CDs-F2 were in good agreement with the simulated results [31]. The morphology of the CDs-F2 sample was studied by scanning electron micrograph (SEM). As shown in Figure S5, the crystal of the CDs-F2 samples exhibits an octahedral structure as reported in UiO-66-based MOFs [24], indicating that the addition of CDs and Eu^3+^ did not change the microstructure of the sample. In addition, with careful observation, the XRD pattern of the CDs-F2 sample appears weak and has broad peaks in the range of 20–40°. This wide peak was speculated to be derived from CDs (Figure 2b), indicating that CDs were successfully incorporated into the F1 material [32]. The FT-IR spectra of the CDs, F1 and CDs-F2 are shown in Figure S6. The FT-IR spectrum of CDs demonstrates that strong peaks related to O-H and N-H appear at 3400 cm^−1^, while peaks at 1437 cm^−1^ and 1374 cm^−1^ are attributed to the typical stretching vibration band of C-N and C-N=, respectively. Additionally the peak at 761 cm^−1^ was ascribed to N-H oscillation vibration [33,34]. CDs with a large number of functional groups on the surface have the potential to synergize with Eu^3+^ [35]. Moreover, F1 shows an absorption peak at 1710 cm^−1^, which is derived from the protonated form of -COOH, indicating that F1 contains free carboxyl groups [36,37]. After Eu(NO_3_)_3_·6H_2_O was introduced into F1, the absorption peak disappeared, implying that Eu^3+^ can be encapsulated in the material and coordinated with -COO^−^. To further confirm the successful introduction of Eu^3+^ into the F1 material, XPS tests were performed. As shown in Figure 2c, compared with F1, CDs-F2 exhibited a new Eu peak at 1100–1200 eV, which confirms that Eu^3+^ was successfully introduced to the F1 material. Meanwhile, the two binding energies of Eu 3d in the CDs-F2 sample located at 1137.6 eV and 1167.3 eV shift in contrast with that of Eu(NO_3_)_3_·6H_2_O (1137.1 eV and 1166.8 eV, respectively) (Figure S7), further indicating that Eu^3+^ was introduced into the material and coordinated with the free carboxyl group of the ligand [38]. Similarly, the N_2_ adsorption measurement was carried out, as shown in Figure 2d. This result reveals the surface area and pore volume of the F1 is 580 m²/g and 0.24 cm³/g, respectively. However, after modification, the surface area and pore volume of CDs-F2 decreased to 455 m²/g and 0.19 cm³/g, respectively. It was demonstrated again that Eu^3+^ and CDs have been introduced into the pore channel of F1. In addition, the energy-dispersive X-ray analysis (EDX) spectrum is demonstrated in Figure S8. Peaks of elements Zr, Eu, C, O, and N can be detected (besides the element Au and partial C from measurement), which confirmed that the Eu^3+^ and CDs were captured in F1. Based on the above results, CDs-F2 with red and blue double emission has been successfully synthesized.

### 3.3. Fluorescence Sensing for Cu^2+^

**Figure 3:**
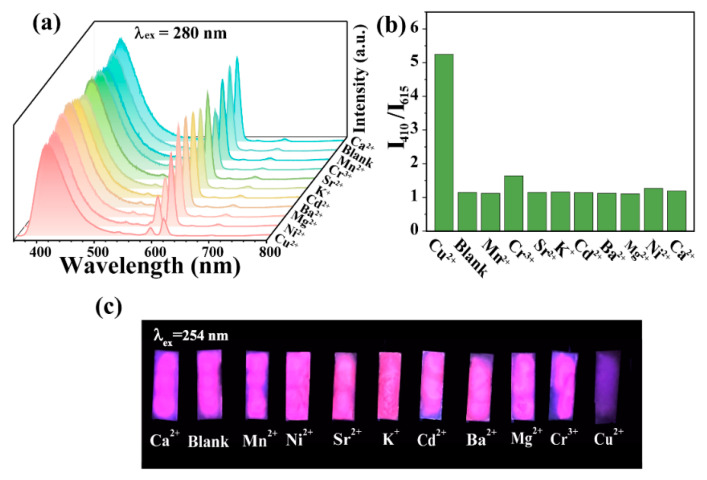
Specific identification performance of CDs-F2 (**a**) and detailed drawing of the selectivity (**b**). (**c**) The photographs of CDs-F2 silica plate containing various metal ions solution under 254 nm UV light irradiation.

Considering the remarkable fluorescence properties of CDs-F2, the potential sensing ability of CDs-F2 for metal ions in an aqueous solution was investigated. Selectivity is an essential factor for fluorescent probes. The fluorescence spectra of CDs-F2 in the presence of different metal ions (Ca^2+^, Mn^2+^, Ni^2+^, Sr^2+^, K^+^, Cd^2+^, Ba^2+^, Mg^2+^, Cr^3+^, Cu^2+^) are illustrated in Figure 3a. It can be found that after adding different metal ions, the blue emission from CDs at 410 nm is almost unchanged, but the characteristic red emission from Eu^3+^ varies with different ions. The most remarkable one is the solution treated by Cu^2+^, the intensity ratio of blue and red emission (I_410 nm_/I_615 nm_) is significantly increased (Figure 3b). This result demonstrates that CDs-F2 can act as a ratiometric fluorescence probe for selectively detecting Cu^2+^ among various metal ions, which can effectively avoid environmental interference. In order to obtain more intuitive detection results, silica plates containing CDs-F2 material were prepared. Interestingly, the selective recognition of Cu^2+^ by the fluorescent probe silica plate was clearly distinguishable to the naked eye, resulting in the luminescence transforming from reddish-purple to blue under 254 nm UV light irradiation (Figure 3c). Anti-interference ability is another important aspect of the performance of a fluorescent probe. Competitive experiments were conducted by monitoring the luminescence intensity of CDs-F2 toward coexisting metal ions in the presence and absence of Cu^2+^. It can be seen that the response of CDs-F2 is not influenced by the coexisting metal ions (Figure S9). When Cu^2+^ is added to the solution, the characteristic emission intensity of CDs (410 nm) in the CDs-F2 materials shows slight variation and that of Eu^3+^ (615 nm) exhibits a significant decrease, whether other metal ions exist or not. It is demonstrated that the CDs-F2 material has excellent anti-interference performance for the recognition of Cu^2+^.

**Figure 4:**
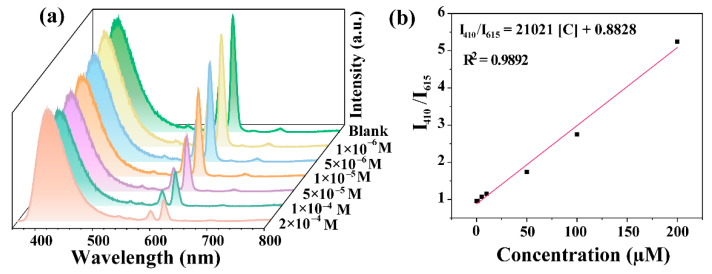
(**a**) The emission spectrum of CDs-F2 with Cu^2+^ concentration. (**b**) The linear relationship between fluorescence emission intensity ratio I_410 nm_/I_615 nm_ and Cu^2+^ concentration.

Sensitivity is one of the key factors determining the further application of fluorescent probes in practical applications [39]. The sensitivity of this probe to Cu^2+^ was determined by measuring the fluorescence intensity ratio (I_410 nm_/I_615 nm_) of CDs-F2 in aqueous solutions of Cu^2+^ with a concentration range of 1 × 10^−6^ to 2 × 10^−4^ M (Figure 4a). The Eu^3+^ characteristic peaks of CDs-F2 weakened sequentially with the increase in Cu^2+^ concentration, while the characteristic peaks of CDs remained stable. As shown in Figure 4b, I_410 nm_/I_615 nm_ exhibited a well-defined linear relationship with the concentration of Cu^2+^. The correlation equation is I_410 nm_/I_615 nm_ = 21021[C] + 0.8828 (R^2^ = 0.9892). The detection limit for Cu^2+^ was calculated as 0.409 µM according to the *3*σ IUPAC standard formula (3σ/K), where σ is the standard deviation of 21 repeated blank tests and K is the slope of the linear equation [16]. This value is much lower than the toxicity level for Cu^2+^ drinking water set by EPA (20 µM) and GB 5749-2022 (15 µM) [40].

Furthermore, the detection performance of the materials in this study for Cu^2+^ in comparison with other works is listed in Table 1. It could be noticed that the present work exhibits a wide linear relationship and low detection limit response compared to our previous work [41] and the other reported fluorescent probes. All the above evidence indicated that CDs-F2 materials are expected to be applied for rapid and immediate detection of Cu^2+^ in aqueous solutions on site.

### 3.4. Possible Sensing Mechanism of CDs-F2 for Cu^2+^

**Figure 5:**
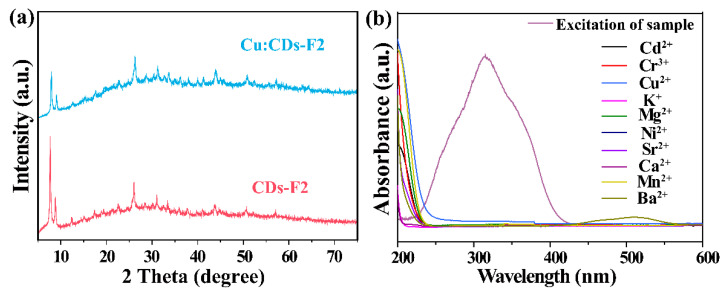
(**a**) PXRD patterns of CDs-F2 before and after sensing the solution of Cu^2+^. (**b**) UV spectra of heavy metals and excitation spectrum of CDs-F2.

**Figure 6:**
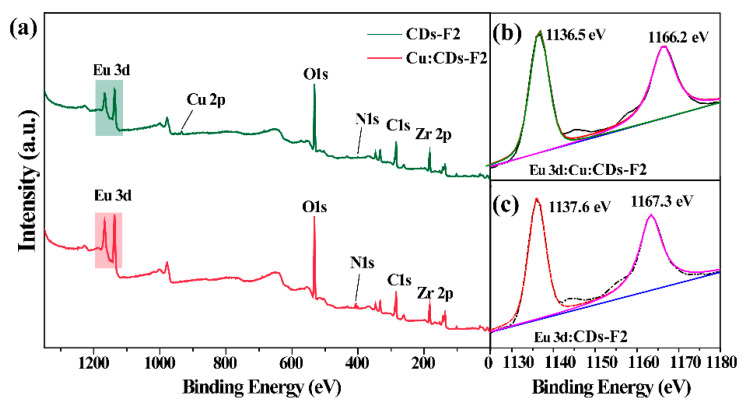
(**a**) XPS spectra of CDs-F2 and Cu: CDs-F2. The binding energy of Eu 3d in Cu: CDs-F2 (**b**) and CDs-F2 (**c**).

Furthermore, the possible mechanism of CDs-F2 for Cu^2+^ detection has been studied. In this study, the mechanism may be attributed to the following two reasons [32]: (i) Cu^2+^ induced the framework collapse; (ii) energy transfer between the Cu^2+^ and the composite. A series of experiments were conducted to gain more insight into the possible quenching mechanism. The XRD pattern of CDs-F2 powder after sensing Cu^2+^ (named Cu: CDs-F2) was first collected in sequence to check the crystal structure. As shown in Figure 5a, it can be seen that it is consistent with the XRD diffraction peak of CDs-F2 material, which proves the structure of the CDs-F2 sample remained the same after being treated with Cu^2+^. Figure 5b shows the excitation spectra of CDs-F2 and the UV absorption spectra of metal ions. The excitation spectra of the probe did not overlap with the excitation spectra of the metal ions, which ruled out the possibility of fluorescence quenching caused by energy transfer between the analyte and the probe [50]. Generally, the fluorescence quenching caused by the formation of non-luminescent intermediates between the fluorophore and the quenching agent is static quenching. In contrast, the fluorescence quenching caused by the collision between the excited fluorophore and the quenching agent is dynamic quenching [51]. To explore whether it is dynamic quenching or static quenching, we studied the fluorescence lifetime of CDs-F2 before and after adding Cu^2+^ (Figure S10). The fluorescence lifetime of CDs-F2 does not change at 410 nm in the presence or absence of Cu^2+^ (0.0104 µs and 0.0103 µs). However, the fluorescence lifetime of the material shortens significantly at 615 nm (260 µs to 19.6 µs). This result suggests that Eu^3+^ and Cu^2+^ occurred dynamic quenching during the sensing process. Subsequently, X-ray photoelectron spectroscopy (XPS) analysis was performed. It can be observed from Figure 6 the binding energies of the Eu 3d orbitals changed from 1137.6 eV and 1167.3 eV to 1136.5 eV and 1166.2 eV, respectively. It may be due to the Cu^2+^ possessing an unsaturated 3d9 electron configuration and a lower metal-centered energy level formed by partially filled d orbitals. The d-d transitions between these energy levels are non-emitting and lead to strong reabsorption, which degrades the luminescence of Eu^3+^ [52].

## 4. Conclusions

In summary, the CDs-F2 fluorescent probe with blue and red double emission was successfully prepared through a one-pot post-synthetic method. The CDs-F2 ratiometric fluorescent probe with self-calibration ratio analysis provides more reliable sensing results. The results demonstrate that the developed CDs-F2 can specifically recognize Cu^2+^ and show excellent anti-interference performance when other metal ions coexist. Meanwhile, silica gel plate fluorescent probes were prepared for fast and visual detection of Cu^2+^. The changed emission color of CDs-F2 in the presence of Cu^2+^ can be easily monitored by the naked eye. Moreover, the sensing mechanism of CDs-F2 for Cu^2+^ detection was systematically investigated. The result reveals that adding Cu^2+^ would affect the energy transfer between the ligand and Eu^3+^, which would quench the luminescence of Eu^3+^. This finding indicates that CDs-F2 material can be employed as a fluorescent probe to rapidly and efficiently detect Cu^2+^ in aqueous solutions. At present, the fluorescent probes we prepared have not been put into practical application. In the follow-up study, it is hoped that the practical application of CDs-F2 can be explored.

## Figures and Tables

**Table 1:** Comparison of LOD and line range of CDs-F2 with other probes for Cu^2+^ detection.

Material	LOD (μM)	Line Range	Ref.
{[Mg_3_(ndc)_2.5_(HCO_2_)_2_(H_2_O)][NH_2_Me_2_] 2H_2_O·DMF}	0.56	10–45 µM	[19]
Eu(FBPT) (H_2_O) (DMF)	8.5	0–17 equiv	[42]
2,4,6-trihydroxybenzaldehyde rhodamine B hydrazone	0.48	0–12 µM	[43]
SF@AgNPs	0.333	1–6 µM	[44]
APA-Rh	1.04	0–40 µM	[45]
Na(Yb,Nd)F_4_@Na(Yb,Gd)F_4_:Tm@NaGdF_4_	0.1	0.125–3.125 µM	[46]
MOF-525 NPs	3.5	1.0–250 nM	[47]
FDPP-C8; TDPP-C8	65 × 10^3^ 127 × 10^3^	0–4 µM; 0–8 µM	[48]
BOPHY-PTZ	----	0–2 µM	[49]
Tb-MOFs	10	1–5 × 10^3^ µM	[41]
CDs-F2	0.409	0–200 µM	This work

## Data Availability

Not applicable.

## Supplementary Materials

The following supporting information can be downloaded at: https://www.mdpi.com/article/10.3390/ma15227933/s1, Scheme S1. Schematic diagram of the preparation process and application of CDs-F2. Figure S1. (a) PL spectra of F1. (b) Emission of F2 with different doped content of Eu^3+^. Figure S2. (a) PL excitation spectra of the CDs. (b) Steady-state emission spectra of CDs at different excitation wavelengths. The steady-state emission spectrum of CDs in the absence and presence of different concentrations of Eu^3+^ (c) λ_ex_ = 360 nm, (d) λ_ex_= 250 nm. Figure S3. Emission of synthetic materials in different proportions. Figure S4. (a) Excitation spectra of CDs-F2 monitored at 615 nm and 410 nm, respectively. (b) Emission of CDs-F2 with excitation wavelengths from 260 to 310 nm. Figure S5. SEM image of CDs-F2. Figure S6. FT-IR spectra analysis of F1, CDs-F2, and CDs. Figure S7. Eu 3d XPS spectra of Eu(NO_3_)_3_·H_2_O and CDs-F2. Figure S8. EDX spectra of CDs-F2. Figure S9. The response of CDs-F2 toward coexisting metal ions in the presence and absence of Cu^2+^: (a) λ_em_ = 410 nm; (b) λ_em_ = 615 nm. Figure S10. Fluorescence lifetimes of CDs-F2 in the absence (a,c) and presence (b,d) of Cu^2+^ in aqueous solution. Table S1. CDs-F2 determined by energy-dispersive analysis by X-rays (EDX).
