# Supplementary material for: A Novel Dual-Emission Fluorescence Probe Based on CDs and Eu3+ Functionalized UiO-66-(COOH)2 Hybrid for Visual Monitoring of Cu2+
Source: Materials (Basel). 2022 Nov 10;15(22):7933. doi: 10.3390/ma15227933 (PMC9692640; doi:10.3390/ma15227933)
Supplement: Supplementary file 1 [file materials-15-07933-s001.zip › materials-2009113-supplementary.pdf]

Supporting Information

A novel dual-emission fluorescence probe based on CDs and Eu^{3+} functionalized UiO-66-$(COOH)_2$ hybrid for visual monitoring Cu^{2+}

Jie Che [1], Xin Jiang [1], Yangchun Fan [1], Mingfeng Li [1], Xuejuan Zhang [2], Daojiang Gao [1], Zhanglei Ning [1,*] and Hongda Li [3,*]

[1] College of Chemistry and Materials Science, Sichuan Normal University, Chengdu 610068, China
[2] The Experiment Center, Shandong Police College, Jinan 250014, China
[3] Liuzhou Key Laboratory for New Energy Vehicle Power Lithium Battery, School of Electronic Engineering, Guangxi University of Science and Technology, Liuzhou 545006, China
* Correspondence: zlning@sicnu.edu.cn (Z.N.); hdli@gxust.edu.cn (H.L.); Tel.: +86-28-84760802 (Z.N.)

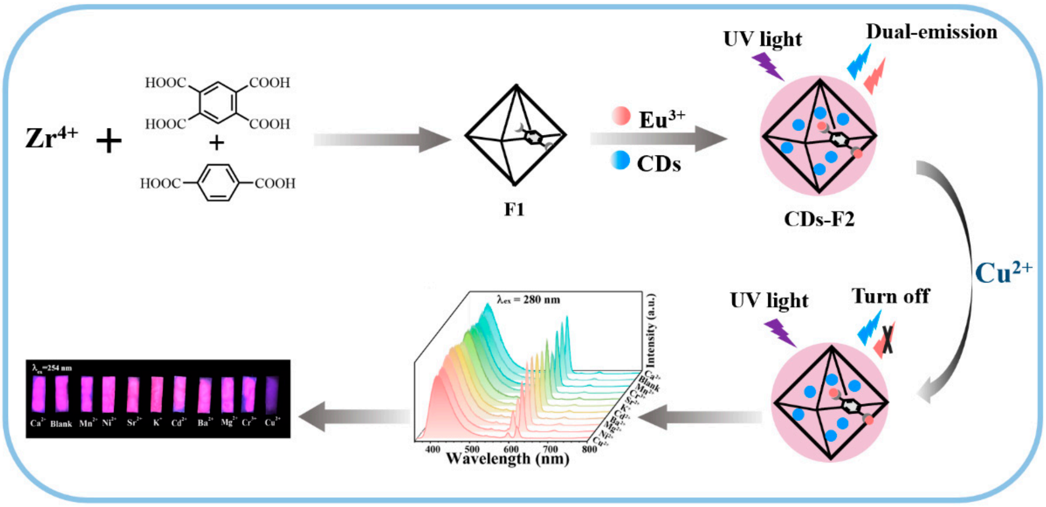

Scheme S1. Schematic diagram of the preparation process and application of CDs-F2.

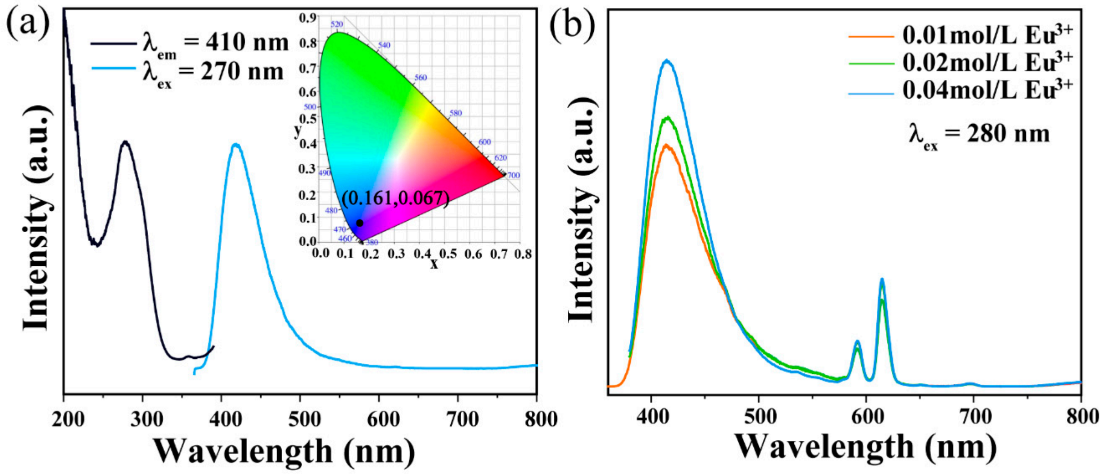

Fig. S1 (a) PL spectra of F1; **(b)** Emission of F2 with different doped content of Eu^{3+}.

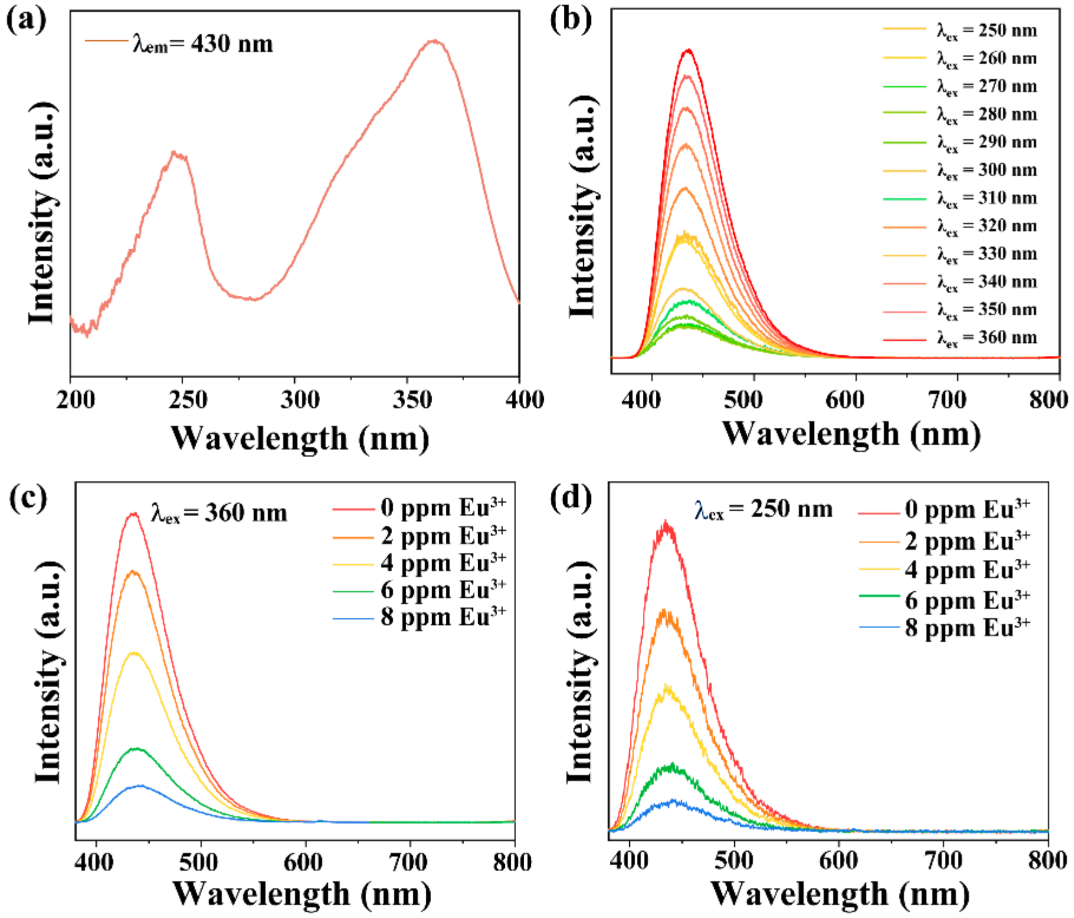

Fig. S2 (a) PL excitation spectra of the CDs; **(b)** Steady-state emission spectra of CDs at different excitation wavelengths; Steady-state emission spectrum of CDs in the absence and presence of different concentrations of Eu^{3+} **(c)** λ_{ex} = 360 nm, **(d)** λ_{ex}= 250 nm.

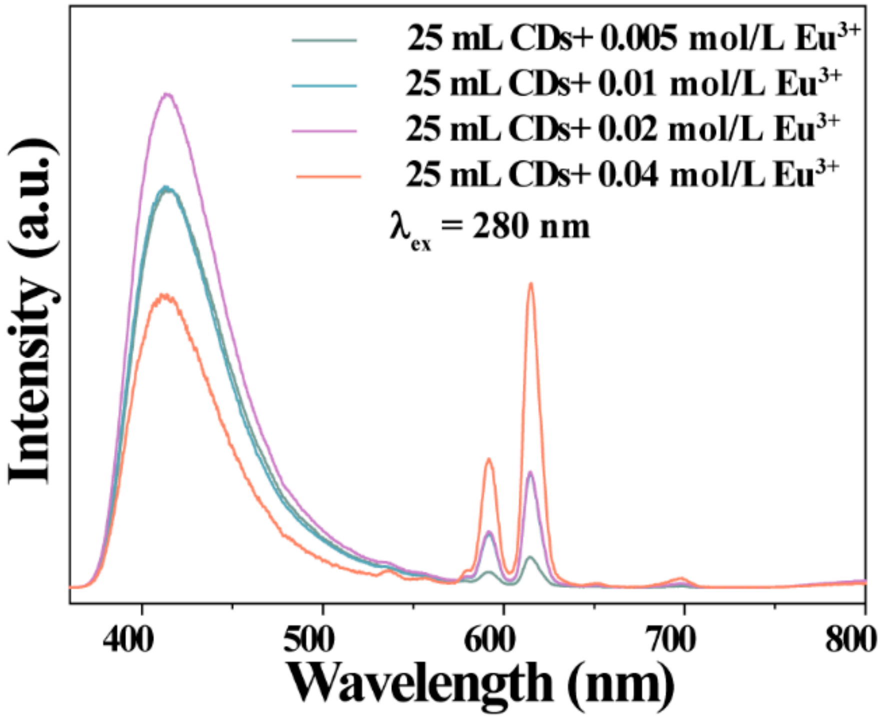

Fig. S3 Emission of synthetic materials in different proportion.

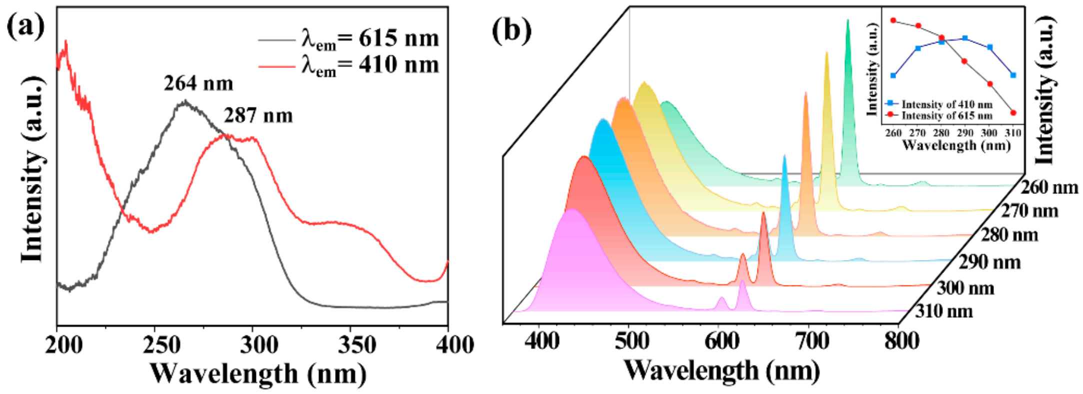

Fig. S4 (a) Excitation spectra of CDs-F2 monitored at 615 nm and 410 nm, respectively. **(b)** Emission of CDs-F2 with the excitation wavelength from 260-310 nm.

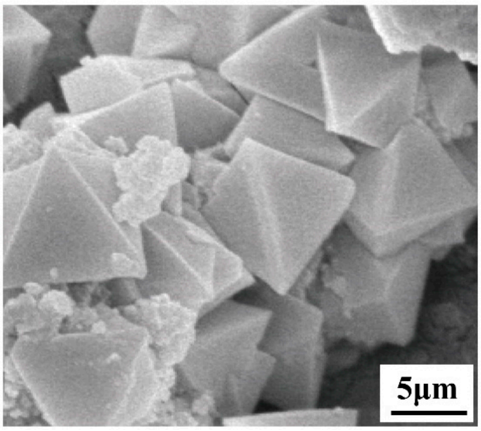

Fig. S5 SEM image of CDs-F2.

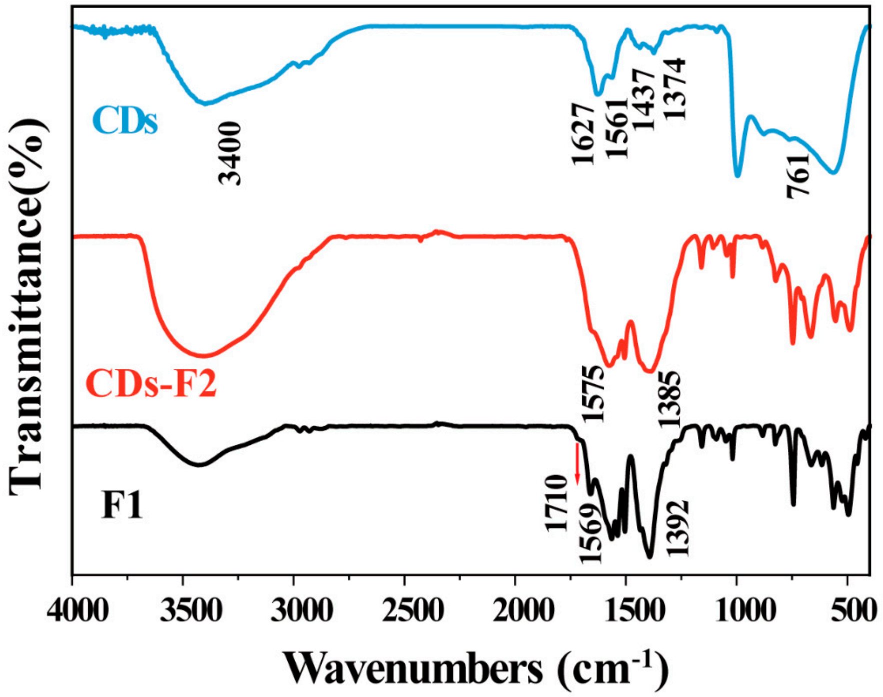

Fig. S6 FT-IR spectra analysis of F1, CDs-F2 and CDs.

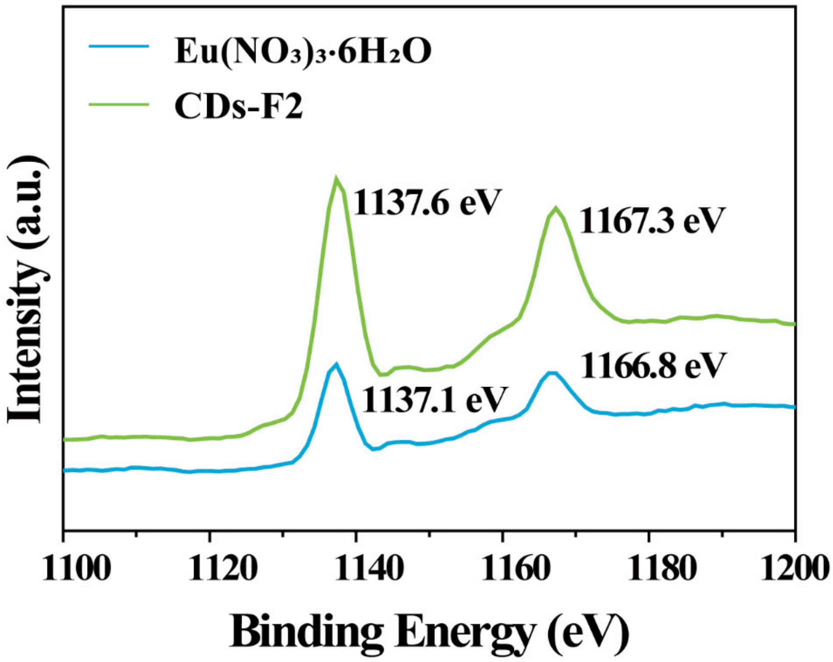

Fig. S7 Eu 3d XPS spectra of $Eu(NO_3)_3{\cdot}H_2O$ and CDs-F2.

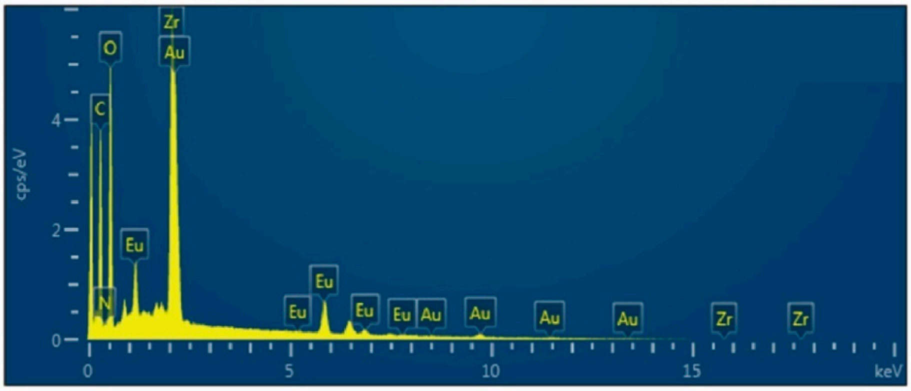

Fig. S8 EDX spectra of CDs-F2.

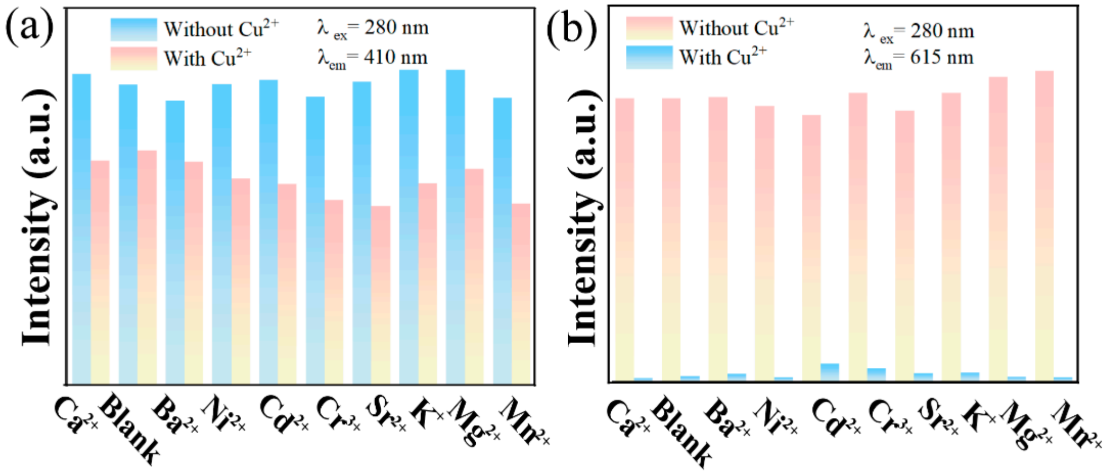

Fig. S9 The response of CDs-F2 toward coexisting metal ions in the presence and absence of Cu^{2+}: **(a)** λ_{em} = 410 nm; **(b)** λ_{em} = 615 nm.

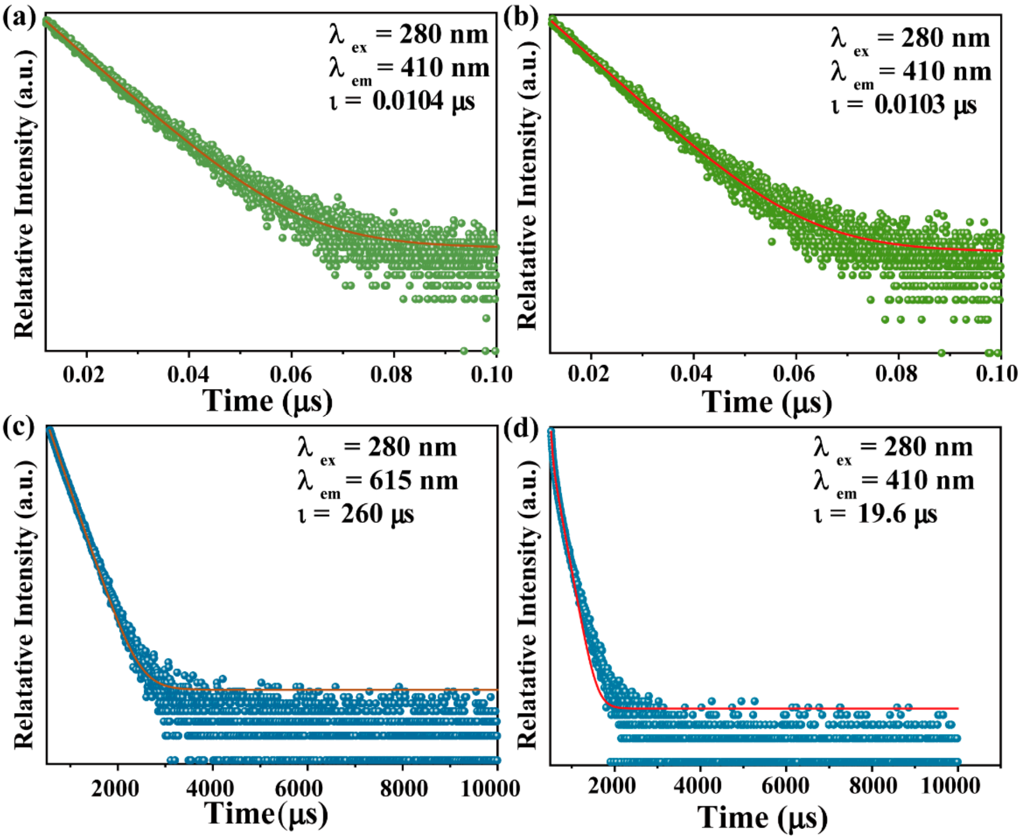

Fig. S10 Fluorescence lifetimes of CDs-F2 in the absence **(a)**, **(c)** and presence **(b)**, **(d)** of Cu^{2+} in aqueous solution.

Table S1 CDs-F2 determined by Energy dispersive analysis by X-rays (EDX).

**Element**	**C**	**N**	**Zr**	**Eu**
Atomic %	56.15	0.78	6.16	0.38
